# Organosulfide-plasticized solid-electrolyte interphase layer enables stable lithium metal anodes for long-cycle lithium-sulfur batteries

**DOI:** 10.1038/s41467-017-00974-x

**Published:** 2017-10-11

**Authors:** Guoxing Li, Yue Gao, Xin He, Qingquan Huang, Shuru Chen, Seong H. Kim, Donghai Wang

**Affiliations:** 1 Department of Mechanical and Nuclear Engineering, The Pennsylvania State University, University Park, PA 16802 USA; 2 Department of Chemical Engineering, The Pennsylvania State University, University Park, PA 16802 USA

## Abstract

Lithium metal is a promising anode candidate for the next-generation rechargeable battery due to its highest specific capacity (3860 mA h g^−1^) and lowest potential, but low Coulombic efficiency and formation of lithium dendrites hinder its practical application. Here, we report a self-formed flexible hybrid solid**-**electrolyte interphase layer through co-deposition of organosulfides/organopolysulfides and inorganic lithium salts using sulfur**-**containing polymers as an additive in the electrolyte. The organosulfides/organopolysulfides serve as “plasticizer” in the solid**-**electrolyte interphase layer to improve its mechanical flexibility and toughness. The as-formed robust solid**-**electrolyte interphase layers enable dendrite-free lithium deposition and significantly improve Coulombic efficiency (99% over 400 cycles at a current density of 2 mA cm^−2^). A lithium**-**sulfur battery based on this strategy exhibits long cycling life (1000 cycles) and good capacity retention. This study reveals an avenue to effectively fabricate stable solid**-**electrolyte interphase layer for solving the issues associated with lithium metal anodes.

## Abstract (web summary)

The practical application of lithium metal anodes suffers from the poor Coulombic efficiency and growth of lithium dendrites. Here, the authors report an approach to enable the self-formation of stable and flexible solid-electrolyte interphase layers which serve to address both issues.

## Introduction

Lithium (Li) metal, which has the highest specific capacity (3860 mA h g^−1^), the lowest potential (−3.040 V vs. a standard hydrogen electrode), and low density (0.59 g cm^−3^)^1–6^, has long been considered as the most attractive anode material. However, applications of rechargeable Li metal batteries have been hindered by several issues^4, 7^. Li metal is highly reactive and reacts with electrolytes to instantly form a solid-electrolyte interphase (SEI) layer on the Li surface. Because of its poor mechanical properties, the SEI layer cannot accommodate large volume change of the Li layer and continuously breaks and repairs during cycling. This repeated breakage and repair consumes both the Li metal and the electrolyte, resulting in low Coulombic efficiency (CE)^4^ and leading to a drying up of electrolyte and serious corrosion of the bulk Li^8^. Furthermore, the Li metal protrusions can grow out of breaks in the SEI layer and Li ions may preferentially deposit on these “naked” Li metal protrusions to form Li dendrites, which can cause short circuit and other serious safety hazards.

To reinforce and stabilize the SEI layer on Li metal, a variety of strategies have been developed such as adjusting electrolyte components^9–11^, optimizing electrolyte additives^7, 12–15^, and fabricating an artificial robust protective layer on the Li surface^16–19^. Among these approaches, electrolyte and electrolyte additives are commonly used for surface stabilization of Li metal^6, 15, 20–28^. Carbonate-based electrolyte (such as LiPF_6_ in carbonate solvent) and ether-based electrolyte (such as LiTFSI in ether solvent) are most commonly used electrolytes. Typical SEI layers formed on the Li metal from these electrolytes consist of inorganic Li salts and organic components such as LiF, Li_2_O, Li_2_CO_3_, and RCOOLi, with the detailed SEI composition dependent on the electrolyte (both salt and solvent) and additives^7, 29–34^. The flexibility and toughness of these SEI layers are not optimized to withstand a large mechanical deformation during Li plating/stripping processes, and the SEI layers still break after cycling, leading to a low CE and Li dendrite formation, especially at high deposition capacity. Ionic liquid and highly concentrated electrolytes have been recently developed showing improved Li plating/stripping performance; however, their lower conductivity and higher viscosity can lead to high polarization and low utilization of cathode capacity^10, 35–37^.

The properties of SEI layers on Li metal are also directly related to cathode chemistry^34,38^. In lithium**-**sulfur (Li-S) batteries, which have been considered as a promising high energy storage device and attracted more and more attention in recent years^39–45^, Li metal deterioration is a major barrier to achieve better cycling stability over 500 cycles. The dissolved and shuttled Li polysulfides can participate in formation of the SEI layer by reacting with Li metal to form the inorganic components (Li_2_S/Li_2_S_2_). Growth of Li dendrite is suppressed and cycling efficiency can be improved to some extent^31, 34^. Such SEI layers still have poor mechanical properties and the aforementioned issues and cannot enable long cycling life of Li**-**S cells. Therefore, the ideal SEI layer should be mechanically strong and also flexible to accommodate the volumetric change during Li plating/stripping process. Design and formation of a SEI layer with high uniformity, excellent flexibility, good mechanical property, and stability is essential to achieve high CE and long cycling life in Li metal batteries.

Herein, we demonstrate a stable and flexible SEI layer through self**-**formation of hybrid inorganic/organic Li compounds onto the Li metal. The organic units in the hybrid serve as a “plasticizer” in the SEI layer to improve its flexibility and toughness, while Li**-**containing inorganic units in the hybrid provide Li conductive pathways. Synergistically, the inorganic/organic hybrid promotes formation of more stable and flexible SEI layers that enable uniform Li deposition, greatly improve CE, and suppress growth of Li dendrites. To fabricate this kind of SEI layer, sulfur**-**containing polymers (SCPs) are used as additives in an ether**-**based electrolyte. These polymers contain sulfur chains and organic components that act as a “bridge” to connect different sulfur chains. Therefore, they retain electrochemical properties similar to those of elemental sulfur and can electrochemically generate both inorganic Li salts (Li_2_S/Li_2_S_2_) and organic units (i.e., organosulfides/organopolysulfides) simultaneously to form a stable hybrid SEI layer on Li metal. In particular, poly(sulfur-random-triallylamine) (PST) is identified as a high-performance additive to show that a high average CE of 99% over 400 cycles can be achieved at a current density of 2 mA cm^−2^ with the capacity of 1 mA h cm^−2^, and at higher capacities the average CE achieved can be as high as 98.9% over 220 cycles (2 mA h cm^−2^, 2 mA cm^−2^) and 98.6% over 220 cycles (3 mA h cm^−2^, 2 mA cm^−2^). Using this hybrid SEI strategy for Li metal, we demonstrate a Li**-**S battery exhibiting a long cycling life (1000 cycles) and good capacity retention.

## Results

### Preparation and utilization of SCPs for SEI formation

**Fig. 1:**
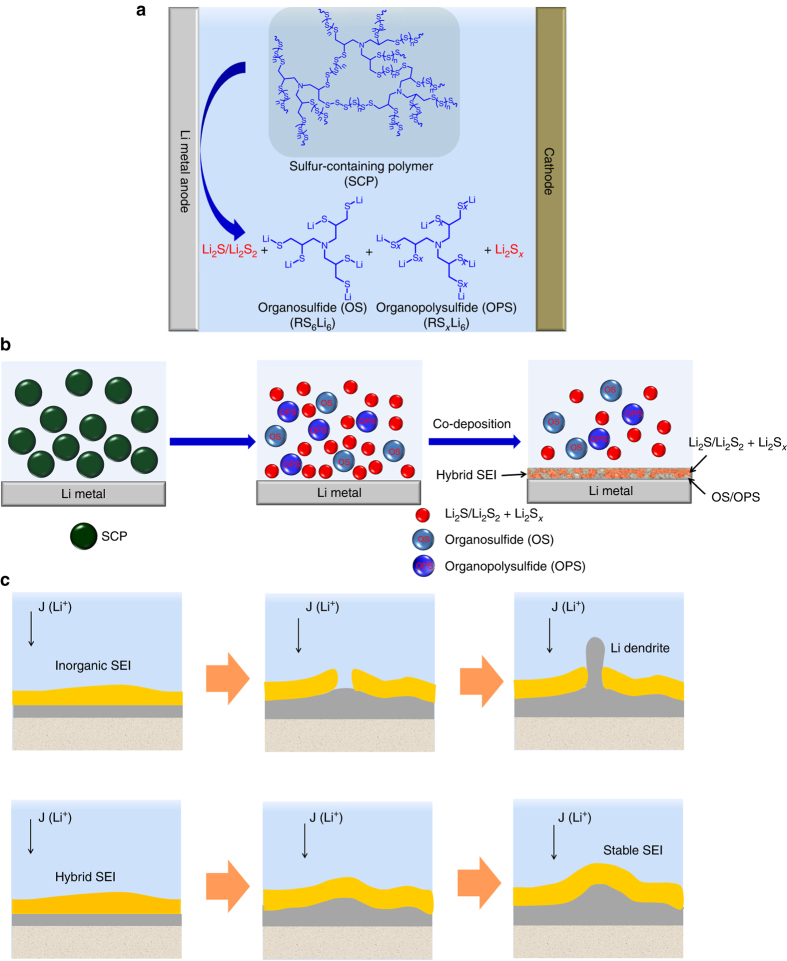
Schematic illustration of the formation of stable inorganic/organic hybrid SEI layer. **a** SCP provides organic units (organosulfide/organopolysulfide) and inorganic units (Li_2_S/Li_2_S_2_) in the electrolyte. **b** Schematics of the formation of organosulfides/organopolysulfides**-**Li_2_S/Li_2_S_2_ hybrid SEI layer. **c** The protection of the Li metal by the stable inorganic/organic hybrid SEI layer

The PST was prepared through a direct copolymerization of sulfur and vinylic monomer triallylamine (TAA). The vinylic monomer was directly dissolved into liquid sulfur followed by heating the molten solution at 145 °C, then sulfur was copolymerized with TAA to obtain PST. PSTs with different sulfur content were synthesized by a variation of feeding ratios of TAA to sulfur during the copolymerization. Specific amounts of PSTs were added in the electrolyte as additive and well mixed. The mixture was then added into the cell and the SCPs were electrochemically decomposed into Li organosulfides (RS_6_Li_6_), Li organopolysulfides (RS_*x*_Li_6_), Li_2_S_*x*_, and Li_2_S/Li_2_S_2_ by contacting Li metal (Fig. 1a, Supplementary Fig. 1). During Li plating/stripping process, an inorganic/organic hybrid SEI layer containing organosulfides/organopolysulfides and Li_2_S/Li_2_S_2_ was self**-**formed on the top of deposited Li metal (Fig. 1b). The organosulfide/organopolysulfide functions as “plasticizer” in the inorganic Li_2_S/Li_2_S_2_ phase to make the hybrid SEI layer more flexible and stable during Li plating/stripping to improve CE and prevent formation of Li dendrites (Fig. 1c).

A coin cell with Li metal electrode and a stainless steel foil (substrate for Li plating/stripping) was used to investigate the process of Li plating/stripping in this work. The electrolyte used here was 1 M LiTFSI (lithium bis(trifluoromethanesulfonyl)imide) and 4 wt% LiNO_3_ in the dioxolane/dimethoxylethane (DOL/DME = 1:1, V/V). The CE of the Li plating/stripping can be calculated from the ratio of Li removed from stainless steel foil to that deposited during the same cycle (as reflected by the total charge for each process)^10, 34^. PST containing 90 wt% sulfur (PST-90) was investigated as the electrolyte additive to study suppression of Li dendrites and enhancement of CE. The electrolyte containing 8 wt% PST-90 was used in the present study (unless otherwise specified) and named PST-90-Electrolyte. Similar studies of Li plating/stripping with the electrolyte containing 8 wt% sulfur (named S-Electrolyte) were also performed to provide a benchmark for comparison, as the sulfur could only provide inorganic units (Li_2_S/Li_2_S_2_) to form the SEI layer during the plating/stripping of Li. In addition, the electrolyte containing higher concentration of LiNO_3_ (4 wt%) in the absence of any sulfur**-**containing compound was also investigated as a control. It is worth noting that increasing the concentration of LiNO_3_ can slightly improve CE and cycling life of Li metal anodes^34^.

### Morphology of the deposited Li metal

**Fig. 2:**
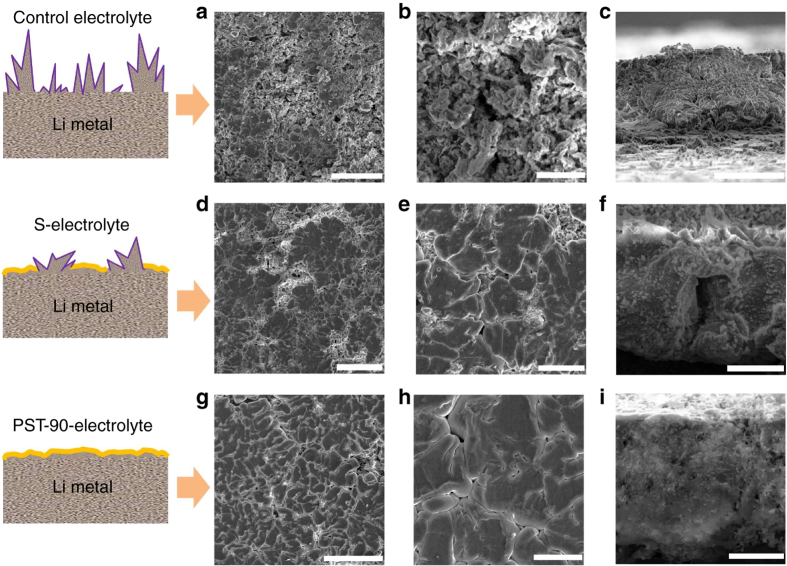
Morphologies of Li metal deposited onto stainless steel substrates. SEM images of Li metal deposited onto bare stainless steel substrates in the control electrolyte (**a**–**c**), S-Electrolyte (**d**–**f**), and PST-90-Electolyte (**g**–**i**) at a current density of 2 mA cm^−2^ and a deposition capacity of 2 mA h cm^−2^. Scale bars in **a**, **b**, **c**: 50, 10, and 10 µm. Scale bars in **d**, **e**, **f**: 50, 10, and 10 µm. Scale bars in **g**, **h**, **i**: 50, 10, and 5 µm. The data shows dense and dendrite**-**free Li made of highly packed Li is obtained after 10 cycles using PST-90-Electrolyte

Morphologies of the deposited Li metal in presence of PST show uniform dendrite**-**free deposition of highly packed Li. Figure 2 shows scanning electron microscopy (SEM) images of Li metal deposited onto bare stainless steel substrates after 10 cycles for the control electrolyte (1 M LiTFSI + 4 wt% LiNO_3_/DOL + DME) (Fig. 2a–c), S-Electrolyte (Fig. 2d–f), and PST-90-Electrolyte (Fig. 2g–i), at a current density of 2 mA cm^−2^ and a deposition capacity of 2 mA h cm^−2^. It is observed that, when S-Electrolyte was used, the deposited Li has a pancake**-**like morphology with relatively low surface area, but the film made of pancake**-**like Li is not continuous and has cracks where the mossy and fluffy Li still grow (Fig. 2d–f). The cross**-**section view also shows that some mossy and fluffy Li grow between the pancake**-**like Li (Fig. 2f). For the PST-90-Electrolyte, dense and dendrite**-**free Li made of highly packed Li is obtained, as visualized in Fig. 2g–i. The cross**-**section view further consolidates that the deposited Li grows very compact and no obvious dendritic Li is observed at the surface and interior of the Li film (Fig. 2i). After 100 cycles at a current density of 2 mA cm^−2^ and a deposition capacity of 2 mA h cm^−2^, using PST-90-Electrolyte, the deposited Li still exhibits very smooth and uniform surface (Supplementary Fig. 2c) and morphological compactness without any growth of Li dendrites (Supplementary Fig. 2f). In contrast, when S-Electrolyte was used, there are many cracks in the deposited Li film (Supplementary Fig. 2b), and dendritic and mossy Li still grows between these cracks after 100 cycles (Supplementary Fig. 2e). To further compare, the deposited Li formed in the control electrolyte bears a large amount of typical dendritic and fluffy structure at both top and cross**-**section views (Fig. 2a–c; Supplementary Fig. 2a, d).

### Characterization of SEI layer

**Fig. 3:**
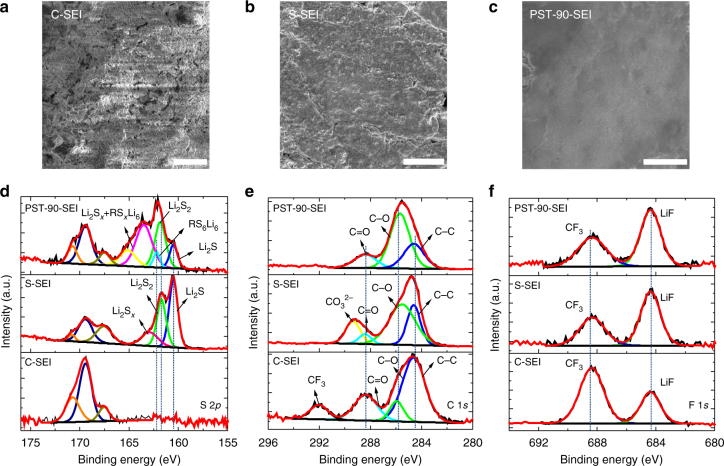
The morphology and XPS spectra of SEI layers formed from the electrolytes containing different additives. **a** SEM image of C-SEI layer. **b** SEM image of S-SEI layer. **c** SEM image of PST-90**-**SEI layer. S 2*p* XPS spectra (**d**), C 1*s* XPS spectra (**e**), and F 1*s* XPS spectra (**f**) of the SEI layers formed from different electrolytes. Scale bar in **a**, **b**, **c**: 10 µm

To explain the continuous, uniform, and dendrite**-**free growth of the Li, properties of the SEI layers after complete Li stripping were characterized. The SEI layers formed from the control electrolyte, the S-Electrolyte, and PST-90-Electrolyte are named C-SEI, S-SEI, PST-90-SEI, respectively. Morphologically, as shown in the SEM images (Fig. 3), the C-SEI layer shows a porous and loose structure, indicating its continuous break during the Li plating/stripping process (Fig. 3a). The S-SEI layer becomes smooth, but cracks are still observed (Fig. 3b), which implies the SEI layer cannot withstand the volume change of Li layer and breaks after the Li plating/stripping process. In contrast, the as**-**formed PST-90-SEI layers show a mostly planar, smooth, and uniform layer with no cracks (Fig. 3c). Such SEI layers can suppress the growth of Li dendrites and thus prevent further contact of Li with the electrolyte, which could correspondingly lead to an enhanced CE.

Chemical characterization of the SEI layers was conducted using Fourier transform infrared (FT-IR) spectroscopy, X-ray photoelectron spectroscopy (XPS) techniques, and Carbon-13 nuclear magnetic resonance (^13^C NMR). Compared with the S-SEI mainly composed of Li_2_S/Li_2_S_2_, FT-IR spectra of PST-90-SEI (Supplementary Fig. 4) shows a peak at ~1170 cm^−1^, which can be found in the pure PST-90 polymer, and is attributed to the vibration of C–N bond. In addition, peaks at 1476 and 838 cm^−1^ are also observed in both PST-90-SEI and PST-90 polymer, indicating the organosulfide/organopolysulfide in the SEI layers originates from PST-90 polymer. Figure 3 shows S 2*p*, C 1*s*, and F 1*s* XPS spectra of different SEI layers. The S 2*p* XPS spectra exhibit major difference among these three SEI layers (Fig. 3d). For the S-SEI, the peaks at 160.5 and 161.7 eV reflect composition of Li_2_S and Li_2_S_2_ in the SEI layer^34, 38^, and the small peak at 163.0 eV is also observed, corresponding to the small amount of Li polysulfides (Li_2_S_*x*_, such as Li_2_S_3_) in the SEI layer^42^. This result demonstrates that the S-SEI layer is mainly composed of inorganic species. For PST-90-SEI layer, besides the peaks at 160.5, 161.7, and 163.0 eV observed in XPS spectra, the additional peak at 162.2 eV corresponding to the S 2*p*_3/2_ from organosulfides is observed, confirming the existence of organosulfides (RS_6_Li_6_) in the SEI layers^46^. The relatively stronger peak at 163.0 eV corresponds to the Li polysulfides (Li_2_S_*x*_) and organopolysulfides (RS_*x*_Li_6_) which have similar position with Li polysulfides^46^. In the C 1*s* XPS spectra (Fig. 3e), the peak at ~292.1 eV can be found in the C-SEI and corresponds to the C 1*s* from the functional group –CF_3_, which may originate from the decomposition of LiTFSI in the control electrolyte^38^. Whereas, this peak disappears when using the PST-90 as the additive in the electrolyte. Moreover, the F 1*s* XPS spectra show two peaks at 684.4 and 688.4 eV assigned to the F 1*s* from the LiF and –CF_3_, respectively (Fig. 3f), and LiF is also the decomposition product of LiTFSI^38^. The intensity of the peak assigned to LiF becomes stronger than that of –CF_3_ when using PST-90 as the additive. Both the C 1*s* and F 1*s* spectra illustrate the –CF_3_ component is suppressed and comparatively the content of LiF increases when using PST-90 as additive. LiF can also facilitate suppressed growth of Li dendrites^47–51^, which have been reported by Archer group. ^13^C NMR was performed to further characterize the components of SEI layers. ^13^C NMR spectra (Supplementary Fig. 6) of organosulfide, which was prepared through the reaction between PST-90 and Li_2_S at a specific ratio in DME, show peaks at 67.48, 64.31, and 48.94 ppm. These peaks are also observed in the sample of PST-90-SEI, indicating the existence of the organosulfide in the hybrid SEI layer. However, for S-SEI and C-SEI, these peaks are absent. Thus, the FT-IR, XPS, and ^13^C NMR spectra support the existence of organosulfides/organopolysulfides in the PST-90-SEI layers, which confirms that the organic units (organosulfides/organopolysulfides) can be co**-**deposited with the inorganic units (Li_2_S/Li_2_S_2_) to form inorganic/organic hybrid SEI film.

**Fig. 4:**
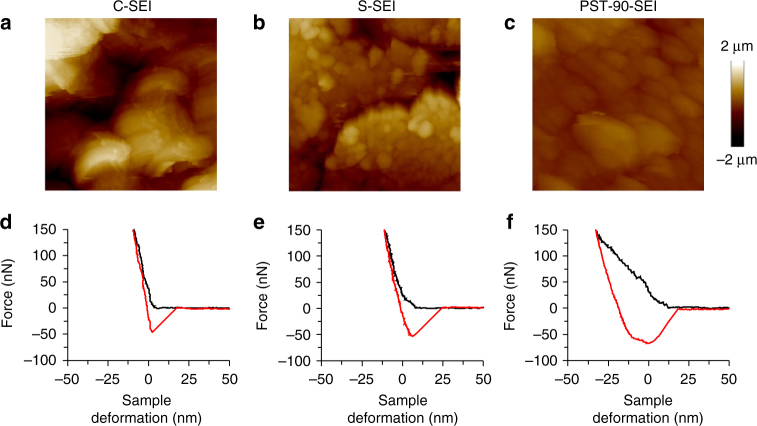
Surface morphology and mechanical property of the SEI layers formed from the different electrolytes. AFM images (10 × 10 µm^2^ scan size) of the C-SEI layer (**a**), S-SEI layer (**b**), and PST-90-SEI layer (**c**). Indentation curves of the C-SEI layer (**d**), S-SEI layer (**e**), and PST-90-SEI layer (**f**). The SEI layers were obtained after 100 cycles of Li plating/stripping, and Li was stripped completely before the AFM characterization

We then examined whether the organic components lead to the change of mechanical properties of the SEI layers. Atomic force microscopy (AFM) was used to investigate mechanical properties of the SEI layers^52–55^. Figure 4a–c displays topographic images of different SEI layers. The C-SEI layer shows large granular features (Fig. 4a), while small particulates are observed everywhere on the surface of S-SEI layer and cracks are also observed (Fig. 4b). In contrast, the PST-90-SEI layers are very smooth with uniform covering, as shown in Fig. 4c. The 3D topographic features observed from AFM results are consistent with the SEM investigation (Fig. 3). Optical profilometry was used to study thickness and coverage degree of the SEI layers. All the SEI layers were obtained after 100 cycles of Li plating/stripping, and Li was stripped completely before the characterization. The results show PST-90-SEI layer has very uniform and low thickness (~10 µm), and the coverage degree is as high as ~95% (Supplementary Fig. 7g–i). In contrast, both C-SEI and S-SEI layer exhibit much higher thickness (~28 µm for C-SEI layer and ~17 µm for S-SEI layer) and lower coverage degree (~50% for C-SEI layer and ~70% for S-SEI layer) (Supplementary Fig. 7a–c and Supplementary Fig. 7d–f). Moreover, the thickness of C-SEI and S-SEI layer is non-uniform, which indicates the continuous cracking of the C-SEI and S-SEI layer during the Li plating/stripping process. The mechanical properties of SEI layers were studied by measuring normal deflection signal change of the cantilever during the tip loading and unloading at the sample surface. Figure 4d–f plot the force as a function of sample deformation, which was calculated by subtracting the cantilever defection from the total piezo drive distance. The cross point of the extrapolated lines of approaching curve and the free**-**standing position curve was set as zero in the indentation position. For C-SEI (Fig. 4d) and S-SEI (Fig. 4e) layers, the slopes of the loading and unloading curves are quite high and overlap each other. This implies that the SEI layers are relatively stiff and viscoelasticity is negligible because these SEI layers are composed of inorganic Li salts, making them more rigid and brittle. In contrast, the surface of PST-90-SEI (Fig. 4g) layer deforms more and shows a large hysteresis between the loading and unloading curves, along with a long pull**-**off or meniscus before the tip completely returned to the free**-**standing position. The reduced modulus of the different SEI layers could be estimated by fitting the unloading curves with the Johnson–Kendall–Roberts (JKR) models (see details of the calculations in Supplementary Note 1 and Supplementary Fig. 8)^56^. The C-SEI and S-SEI layers show the modulus of 903 and 740 MPa from the JKR model fit, respectively. In contrast, PST-90-SEI layer displays a low modulus estimated to be 367 MPa (Supplementary Table 1). These results suggest that the organosulfides/organopolysulfides**-**containing SEI layer (PST-90-SEI) becomes soft and viscoelastic to render it flexible, which is beneficial to withstand the large mechanical deformation originating from the Li plating/stripping and to both suppress the growth of Li dendrite and improve cycling CE.

### Electrochemical performance

**Fig. 5:**
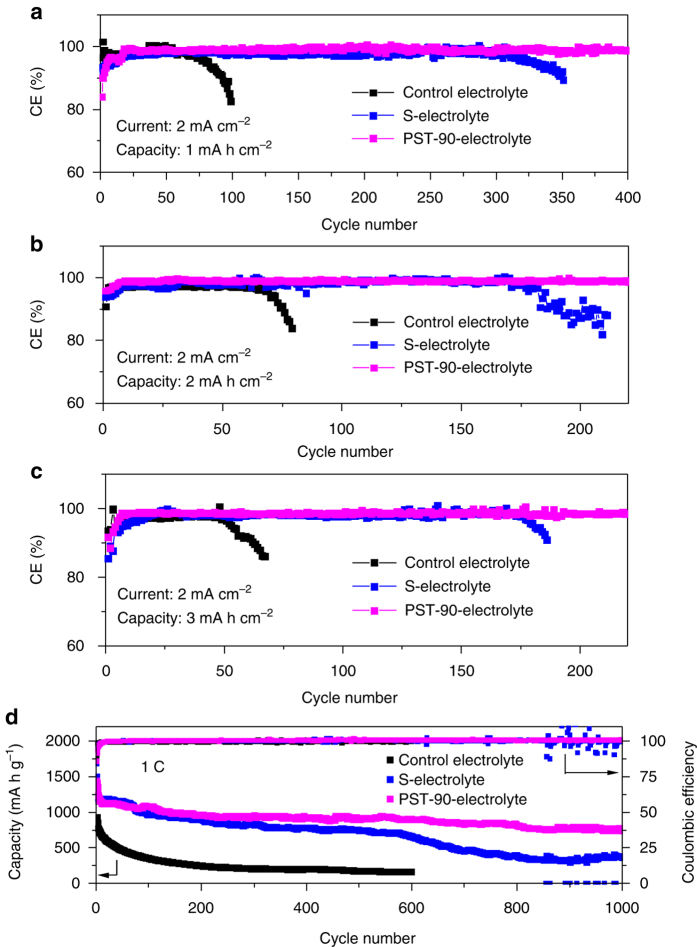
Characterization of electrochemical performance. Cycling performances of the cells using PST-90-Electrolyte (magenta symbols) at a current density of 2 mA cm^−2^ with a deposition capacity of 1 mA h cm^−2^ (**a**); at a current density of 2 mA cm^−2^ with a deposition capacity of 2 mA h cm^−2^ (**b**); at a current density of 2 mA cm^−2^ with a deposition capacity of 3 mA h cm^−2^ (**c**). The black and blue symbols represent data of the samples using the control electrolyte and the S-Electrolyte, respectively. **d** The electrochemical performance of the Li**-**S batteries using electrolytes containing different additives at a rate of 1C. The Ketjen Black (KB) containing 70 wt% S was used as the cathode material. The areal sulfur loading is 1.5 mg cm^−2^

Li plating/stripping CE and cycling stability using PST-90-Electrolyte were studied. Figure 5 shows the cycling performance of cells using the PST-90-Electrolyte at the same current density of 2 mA cm^−2^ and different deposition capacities. Under the deposition capacity of 1 mA h cm^−2^ (Fig. 5a), the cells deliver enhanced average CE of 99% over 400 cycles. When the capacity was increased to 2 mA h cm^−2^ (Fig. 5b), the cells could be stably cycled for 220 cycles with the CE still maintaining a high average value of 98.9%. Notably, when the capacity was further elevated to 3 mA h cm^−2^ (Fig. 5c), a stable cycling performance for 220 cycles was achieved and the cells exhibited an average CE as high as 98.6%. Comparing with those using the control electrolyte or S-Electrolyte, the cells with PST-90-Electrolyte showed much better cycling stability and higher average CE. It is noted that at the deposition capacity of 1 mA h cm^−2^, the initial CE of cells using PST-90-Electrolyte is lower than that of cells using S-Electrolyte. This is because that PST-90 reacts with Li metal much easier and faster than sulfur, which can be reflected by their reduction potentials (Supplementary Fig. 9), and therefore consumes more Li before the formation of uniform and stable SEI layer in the first cycle. The cycling performance of cells using PST-90-Electrolyte at higher current densities was also investigated. The deposition capacity was kept at 2 mA h cm^−2^, when the current density of 3 mA cm^−2^ was applied (Supplementary Fig. 10a), the cells deliver an average CE of 98.7% over 200 cycles. When the current density was elevated to 4 mA cm^−2^ (Supplementary Fig. 10b), a high average CE of 98.6% over 150 cycles could still be achieved. These results demonstrate the hybrid SEI layer is stable at high current densities. In addition, we further evaluated the cycling performance of cells with electrolytes only using sulfur-containing compounds as additives (Supplementary Fig. 11). For the control electrolyte (1 M LiTFSI/DOL + DME) and the electrolyte containing 8% sulfur alone (1 M LiTFSI/DOL + DME + 8% sulfur), the cells exhibit very poor cycling stability and low average CE. However, the cells with electrolyte only containing PST-90 as additive deliver an enhanced average CE of 90.5% over 100 cycles. These results demonstrate the PST-90 alone is still beneficial for the improvement of CE and cycling stability of Li metal anodes, even though the CE is lower than that combined with LiNO_3_. This also, on the other hand, indicates LiNO_3_ has a synergetic effect on the protection of Li metal anodes. LiNO_3_ can passivate the Li surface^57, 58^ and retard the reaction rate between sulfur-containing compounds and Li to enable a mild formation of degradation products, which is beneficial for the more uniform co-deposition of these degradation products to form the stable hybrid SEI layer.

The formation of stable SEI layer could also be reflected by the cell polarization and its evolution with cycling. The voltage profiles (Supplementary Fig. 12a) show that the polarization of the cells using PST-90-Electrolyte is smaller than those with the S-Electrolyte. The voltage hysteresis for the cells using PST-90-Electrolyte has no obvious increase, and remains almost unchanged at ~60 mV over 200 cycles (Supplementary Fig. 12b). The smaller hysteresis is attributed to the stable and flexible SEI layer, which obtained from the co**-**deposition of organosulfides/organopolysulfides and inorganic Li salts (Li_2_S/Li_2_S_2_). This unique SEI layer is robust enough to accommodate the large volume change of the Li layer and make the growth of deposited Li more compact and uniform (as shown in Fig. 2g–i; Supplementary Fig. 2c, f; Fig. 3c). The compact and uniform Li would have smaller surface area leading to much thinner SEI accumulated over the electrode surface during cycles and therefore a much smaller cycling overpotential. In contrast, for the cell with the S-Electrolyte, the voltage hysteresis in the Li plating/stripping first increases as the SEI layer accumulates then remains stable at 105 mV till 160 cycles, but after 160 cycles the voltage hysteresis begins to decrease. This phenomenon could be attributed to the crack of the SEI film and the creation of greater Li surface area, which have been evidenced by the SEM morphologies (Fig. 2d–f; Supplementary Fig. 2b, e; Fig. 3b).

The stable SEI layer formed using PST-90 as the additive in the electrolyte can also be evidenced by electrochemical impedance spectroscopy (EIS). Supplementary Fig. 13 shows the Nyquist plots of the cells using various electrolytes after different cycles of Li plating/stripping. The depressed semicircle in the high**-**to**-**medium frequency region is assigned to the passivating surface layer formed on the current collector^45, 59^. For the cells using the control electrolyte or the S-Electrolyte, a considerable increase in the diameter of the semicircle (Supplementary Fig. 13a, b) is observed as the cycle increases, which indicates the SEI layer on the current collector is accumulated and becomes thicker and thicker. In contrast, for the cells with the PST-90-Electrolyte, there is no obvious change in the semicircle (Supplementary Fig. 13c), which implies the SEI layer formed on the current collector is very stable and the thickness does not change noticeably. The EIS results reconfirm that the SEI layers formed from the PST-90-Electrolyte are much more stable than those formed from the control electrolytes or the S-Electrolyte. Such stable SEI layers, once formed on the surface of Li, can prevent further Li reacting with electrolyte, which leads to an enhanced CE.

Li**-**S batteries were also fabricated using the PST-90 as the additive in the electrolyte. KB containing 70 wt% S was used as the cathode material. All the cells were running at a rate of 1C (1C = 1672 mA g^−1^). Figure 5d shows the electrochemical performance of the Li**-**S batteries. For the cells using the control electrolyte, the capacity drops very fast, after 100 cycles, the capacity decreases to 350 mA h g^−1^ from its initial capacity (1153 mA h g^−1^). When PST-90 is added in the electrolyte, the initial capacity increases to 1431 mA h g^−1^. This is because the PST-90 in the electrolyte contributes extra capacity to the cells (Supplementary Fig. 14). After 10 cycles, the capacity decreases to 1143 mA h g^−1^. The capacity drop at this stage may be ascribed to the consumption of PST-90 to form the SEI layer. The cycling performance is fairly stable from the 10th cycle to the 1000th cycle, and retains a capacity of 735 mA h g^−1^ with CE of ~99.9%. In contrast, when S-Electrolyte was used, the capacity drops faster than that of cells with PST-90 as additive, and after the 600th cycle the capacity drops quickly and the CE exhibits irregular fluctuating behavior. This may be ascribed to the drying up of electrolyte, suggesting Li still reacts with the electrolyte during cycling. The electrochemical performance of the cells reflects that PST-90 in the electrolytes can protect the Li metal anode effectively and enable the stable cycling performance of the sulfur cathode.

## Discussion

As demonstrated above, the hybrid SEI layers are self-formed by co**-**deposition of organosulfide, organopolysulfide, and inorganic Li_2_S/Li_2_S_2_, all of which are released from PST additives. The presence of the organic components increases the viscoelasticity of the SEI layer, the nitrogen atoms in the organic components interact with Li ions, causing them to distribute more uniformly on the surface of electrode than alkyl components, which is beneficial for a more uniform deposition of Li, while inorganic Li_2_S/Li_2_S_2_ provides Li conductive pathway and necessary mechanical hardness in the SEI layer. These multifunctional components endow the SEI layer with unique features. The content ratio between the organic and inorganic components in PST can alter the properties of the hybrid SEI layers and consequently affect Li plating/stripping cycling performance. To investigate this effect, the PSTs with 50 and 70 wt% sulfur (designated as PST-50 and PST-70, respectively) were also prepared and compared with PST-90 in terms of Li plating/stripping performance as the electrolyte additives (Supplementary Table 2). Based on the assumption of complete reduction of the PST into Li_2_S and Li organosulfide, the SEI layers induced by PST-50, PST-70, and PST-90 additives represent those with different sulfur ratios of inorganic component to organic components, which are inorganic-Li_2_S/Li_2_S_2_-free, organosulfide-dominant, and inorganic-Li_2_S/Li_2_S_2_-dominant SEI layers, respectively. The electrolyte used here was 1 M LiTFSI and 1 wt% LiNO_3_ in the DOL/DME = 1:1 (V/V). It is found that the cells using PST-50 or PST-70 as additive exhibit poor cycling performance and the cycling stability drops with the decrease of sulfur content in the PST, as shown in Supplementary Fig. 16. This reflects that the inorganic-Li_2_S/Li_2_S_2_-free and organosulfide-dominant SEI layers are not stable for high efficiency Li plating/stripping, indicating an important role of inorganic component of Li_2_S/Li_2_S_2_ in the hybrid SEI layers to achieve improved performance. This is possibly ascribed to the effectiveness of inorganic Li_2_S/Li_2_S_2_ component in providing Li-ion conductive pathway and necessary mechanical hardness, and thus promoting uniform deposition of Li. However, pure inorganic Li_2_S/Li_2_S_2_ is too brittle to tolerate large volume change during Li plating/stripping and provide uniform Li deposition. Thus, it is advantageous in using PST-90 to form a hybrid SEI with an optimized content that synergizes both inorganic Li_2_S/Li_2_S_2_ and organosulfide in formation of a stable hybrid SEI layer for high-efficiency Li plating/stripping. The small molecules, such as dimethyl disulfide (DMDS) and dimethyl trisulfide (DMTS), which contain short S–S bonds and simple alkyl components (methyl group), were also used as the electrolyte additives and the electrochemical performance was also investigated (Supplementary Fig. 17). The cells with DMDS or DMTS as additive deliver very poor cycling stability and low CE, indicating simple alkyl sulfides originating from DMDS and DMTS could not enable the formation of stable SEI and suppress the electrolyte composition.

The content of PST-90 as additives in the electrolyte also affects the Li plating/stripping cycling performance. Comparing the Li plating/stripping performance of using electrolyte containing 2, 5, 8, and 10 wt% of PST-90, we found that the cycling life is improved with increasing content of PST-90 additive (Supplementary Fig. 18) till reaching 8 wt%. When the content of PST-90 in the electrolyte increased to 10 wt%, the cycling life deteriorates, probably due to increased consumption of Li for formation of Li organosulfides and polysulfides and thicker hybrid SEI layers with decreased conductance.

These results demonstrate that the component content of PST and the content of PST in the electrolyte are both critical to the formation of highly stable hybrid SEI layers. Other SCPs with different organic components could also be used in formation of stable inorganic/organic hybrid SEI layer. It is worth noting that the function of different organic components of SCPs in formation of the hybrid SEI layers is still a challenge to evaluate and requires further investigation. Moreover, other electrochemically active or inactive organic functional components beyond the organosulfides can also be considered as promising candidates to fabricate hybrid SEI layer: (1) Li-ion-affinity organic functional components which have interactions with Li ions can be involved in the SEI layers to guide the distribution of Li ions and in turn lead to a uniform deposition of Li metal. (2) Self-healing organic functional components which endow the SEI layer with self-repairable properties can be used to fabricate self-healing SEI. (3) Additive-like organic functional components involved in the SEI layer can be electrochemically decomposed to enable a much more robust SEI layer.

In conclusion, we have demonstrated a strategy to fabricate stable and flexible SEI layer through self**-**formation of hybrid inorganic/organic Li compounds onto the Li metal. SCPs (PST) can be used as additives in the electrolyte to self**-**form the organic (organosulfides/organopolysulfides) and inorganic components (Li_2_S/Li_2_S_2_). The organosulfides/organopolysulfides can co**-**deposit with insoluble Li salts (Li_2_S/Li_2_S_2_) to form inorganic/organic hybrid SEI layer, in which the organosulfides/organopolysulfides function as “plasticizer” in the inorganic phase to make the SEI layer more flexible and stable. This stable SEI layer is robust enough to accommodate the large volume change of the Li layer, prevent the growth of Li dendrites, and greatly minimize the electrolyte decomposition. Therefore, the growth of deposited Li in our work was much more continuous, uniform and compact, and the CE and cycling stability were also improved. When using PST-90 as the additive, an average CE as high as 99% over 400 cycles was obtained at a current density of 2 mA cm^−2^ with the capacity of 1 mA h cm^−2^. At a practical current density of 2 mA cm^−2^ with higher capacities of 2 and 3 mA h cm^−2^, the high average CE of 98.9 and 98.6% over 220 cycles can be achieved, respectively. Meanwhile, a Li**-**S battery with PST-90 as additive in the electrolyte exhibited long cycling life (1000 cycles) and good capacity retention. Our finding shows that the suitable organic units can function as “SEI plasticizer” to co**-**deposit with the inorganic species to form much more stable and flexible SEI layer. This study provides a new and facile strategy to fabricate effective SEI layer for solving dendrite issues associated with Li metal anodes, which could be beneficial for the safe and highly efficient utilization of Li metal electrodes for advanced energy storage applications.

## Methods

### General procedure for the preparation of PST

To a 24-ml glass vial equipped with a magnetic stir bar was added sulfur and the vial was then heated to 145 °C in a thermostatted oil bath until a clear orange**-**colored molten phase was formed. TAA was then added directly to the molten sulfur medium via a syringe. The resulting mixture was stirred at 145 °C for 8–10 min, which resulted in vitrification of the reaction medium. After allowing the reaction mixture to cool to room temperature, a brown solid (PST) was formed (detailed synthetic and processing protocols are given in the Supplementary Information).

### Electrochemical measurements

Galvanostatic experiments were performed using a Lanhe battery testing station at room temperature. The electrolytes were prepared by mixing the desired amount of salts and additives into the solvent. The process of Li metal plating/stripping was investigated using a two**-**electrode configuration assembled in the 2016**-**type coin cells (MTI Corporation), which is composed of a Li metal electrode and a stainless steel foil (substrate for Li plating/stripping). A constant current is applied to the electrodes, while the potential is recorded vs. time. A fixed amount of Li is first deposited onto the stainless steel substrate followed by subsequent process of Li dissolution and re**-**deposition. CE is calculated by dividing the amount of Li stripped by the amount of Li plated on the stainless steel foil during each cycle. The EIS data were recorded using a Solartron SI1287 Electrochemical Interface by applying a sine wave with an amplitude of 5 mV over a frequency range of 100 kHz–0.1 Hz. Cyclic voltammetry curves were collected using a CHI 660D electrochemical workstation at a scan rate of 0.02 mV s^−1^ from 3.0 to 1.7 V.

KB EC60JD (AkzoNobel) was used as the conductive framework for making C/S composites with 70 wt% sulfur. They were prepared by thermal treatment of the elemental sulfur and KB mixtures at 160 °C in sealed glass vials for 10 h. The composites were combined with conductive Super C and poly(vinylidene fluoride) as a binder in a mass ratio of 80:10:10 and stirred into slurry with N**-**methyl**-**2**-**pyrrolidone. The slurry was then blade cast onto carbon**-**coated aluminum (Al) foil and dried at 50 °C overnight in a vacuum oven to obtain the C/S cathode electrodes. The sulfur**-**free cathode was prepared similarly, with the C/S composite simply being replaced by KB in the slurry. CR2016 coin cells were assembled in an argon**-**filled glove box employing the C/S composite-coated Al foil as the cathode, a Celgard 2325 as the separator, and Li foil as the reference/counter electrode. The electrolyte used was 1 M LiTFSI and 4 wt% LiNO_3_ in the DOL/DME = 1:1 (V/V) with different additives.

### Characterization

SEM observations were performed on SEM (Nano630 FE-SEM). FT-IR was performed on a Bruker Vertex V70 spectrometer in diffuse reflection mode with a Spectra Tech Collector II accessory. XPS measurements were carried out with a Kratos XSAM800 Ultra spectrometer. ^13^C NMR characterization was performed on a Bruker AV-3-HD-500. AFM (Digital Instrument Multimode scanning probe microscope) was used to investigate the surface morphology and analyze the mechanical property of the SEI layer under inert atmosphere. The 3D topographic images of SEI layers were recorded through tapping mode imaging with sharp AFM tips (BRUKER TESPA-V2). The scan size was 10 × 10 μm. Another kind of silicon tip (BudgetSensors Multi75Al-G) was employed to test the mechanical behavior of different SEI layers. The spring constants of the AFM cantilevers were calibrated by the Sader method (around 3.2–3.4 N/m)^60^. All tips were cleaned with UV/ozone to remove organic contaminants. The force**-**distance curve was obtained from AFM indentation test. The maximum indentation force was kept constant at 150 nN for each test and the vertical probe rate was 316 nm/s. Zygo 3D Optical Surface Profilers were used to investigate the thickness and coverage degree of SEI layers under inert atmosphere. The scan size was 450 × 450 μm.

### Data availability

The data that support the findings of this study are available on reasonable request from the corresponding author.

## Electronic supplementary material

Supplementary Information
