# Supplementary material for: Organosulfide-plasticized solid-electrolyte interphase layer enables stable lithium metal anodes for long-cycle lithium-sulfur batteries
Source: Nat Commun. 2017 Oct 11;8:850. doi: 10.1038/s41467-017-00974-x (PMC5636837; doi:10.1038/s41467-017-00974-x)
Supplement: Supplementary file 1 — Supplementary Information [file 41467_2017_974_MOESM1_ESM.pdf]

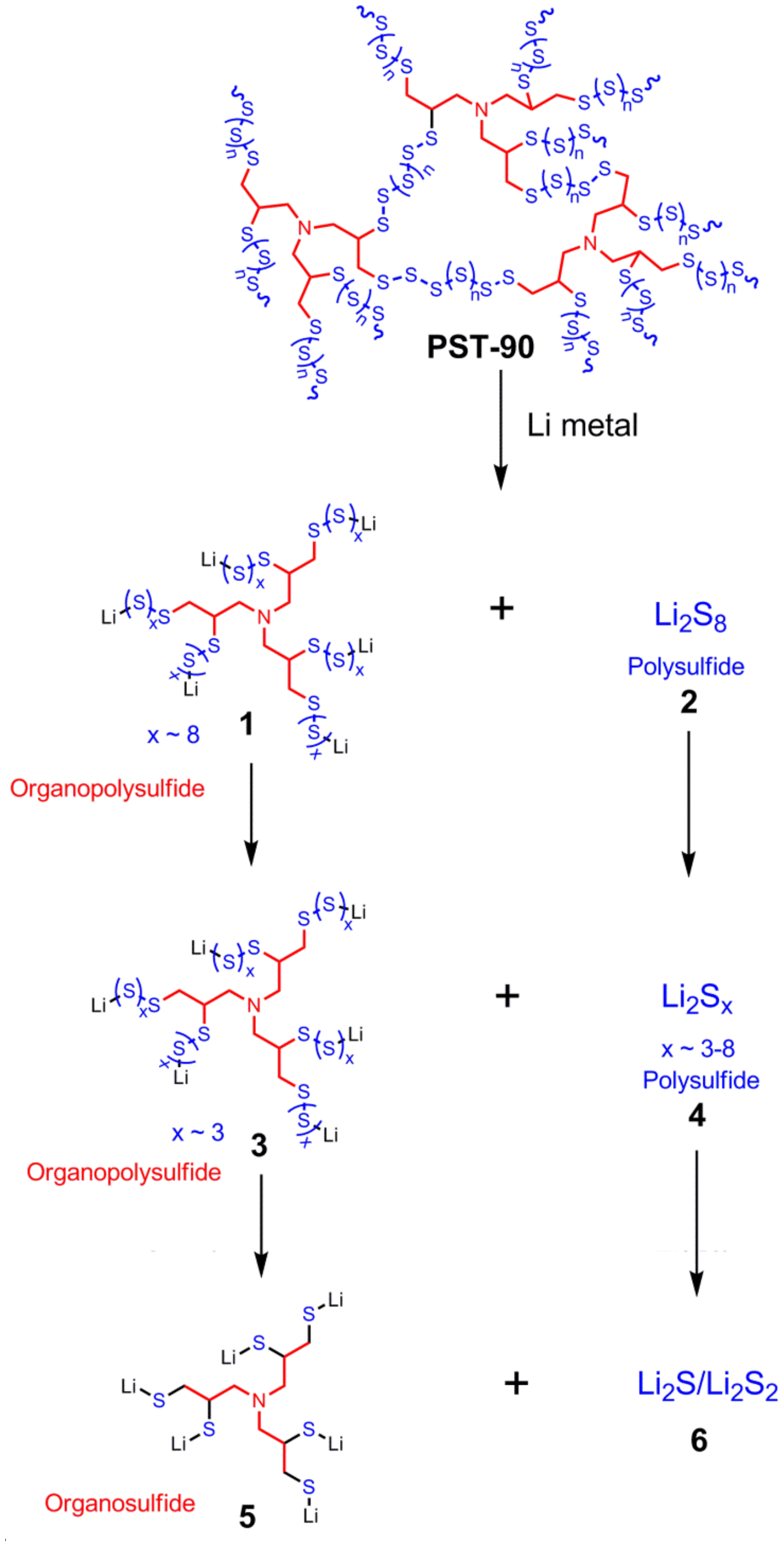

Supplementary Figure 1. Proposed mechanism for the formation of organic components and inorganic Li salts from PST-90. When contacting with Li metal, PST-

90 first generates high order organopolysulfide and Li_2S_8 (1 and 2 in Supplementary Fig. 1), and with further reaction generates low order organopolysulfide and Li_2S_x ($3<x<8$) (3 and 4 in Supplementary Fig. 1). Continued reaction results in the conversion of 3 and 4 into organosulfide and insoluble inorganic Li salts of Li_2S/Li_2S_2 (5 and 6 in in Supplementary Fig. 1), which co-deposit on the Li metal to self-form the stable hybrid SEI layer.

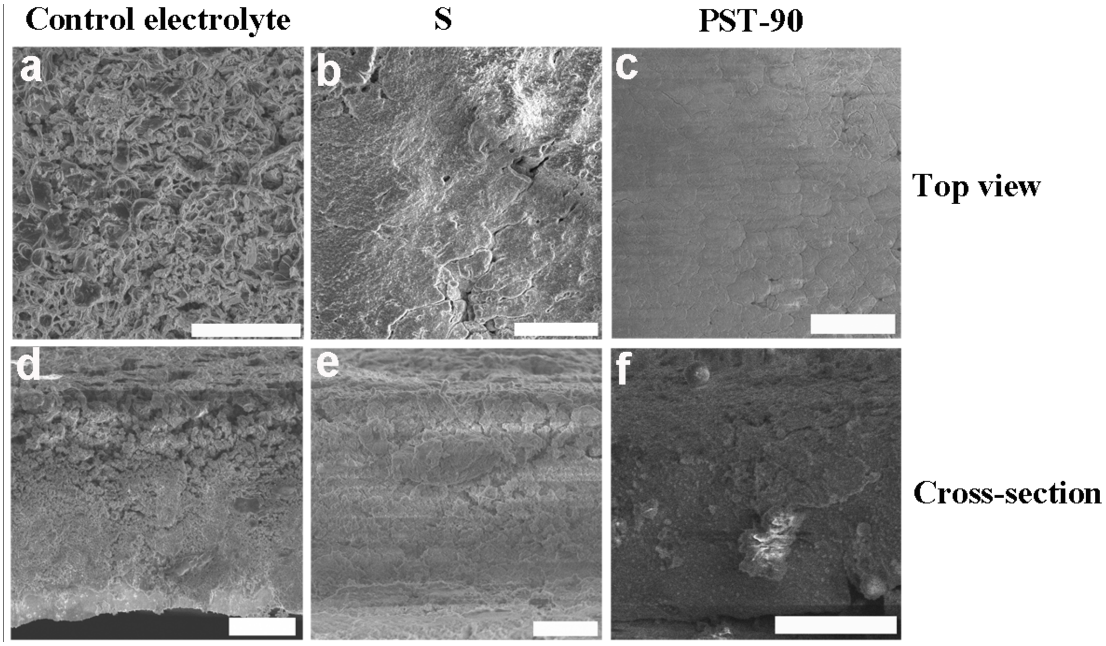

Supplementary Figure 2. SEM images of the deposited Li after 100 cycles at a current density of 2 mA cm^{-2} and a deposition capacity of 2 mA h cm^{-2}. (**a, d**) Top view and cross-section view of deposit Li using the control electrolyte (1 M LiTFSI + 4 wt% $LiNO_3$/DOL + DME). (**b, e**) Top view and cross-section view of deposit Li using the S-Electrolyte. (**c, f**) Top view and cross-section view of deposit Li using the PST-90-Electrolyte. Scale bar in a, b and c: 20 µm. Scale bar in d, e and f: 10 µm.

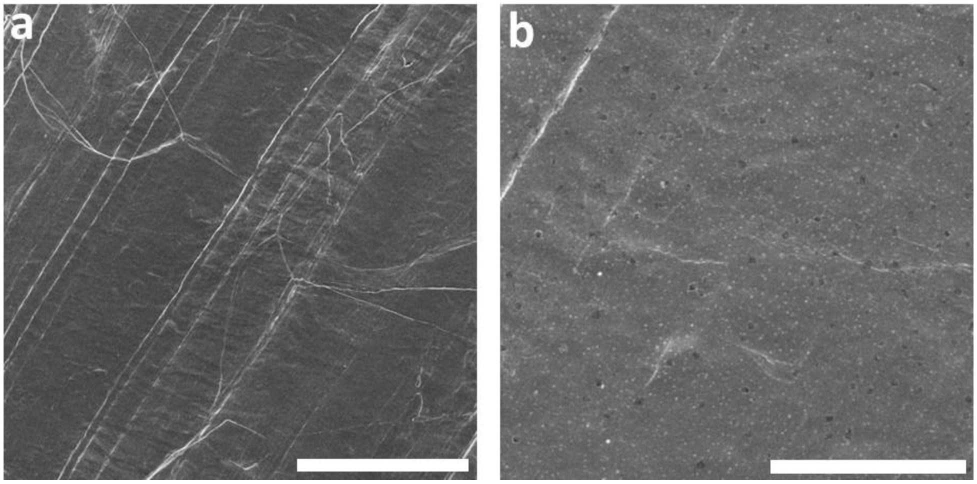

Supplementary Figure 3. SEM images of pristine Li metal foil. Scale bars in a and b: 200 and 20 μm.

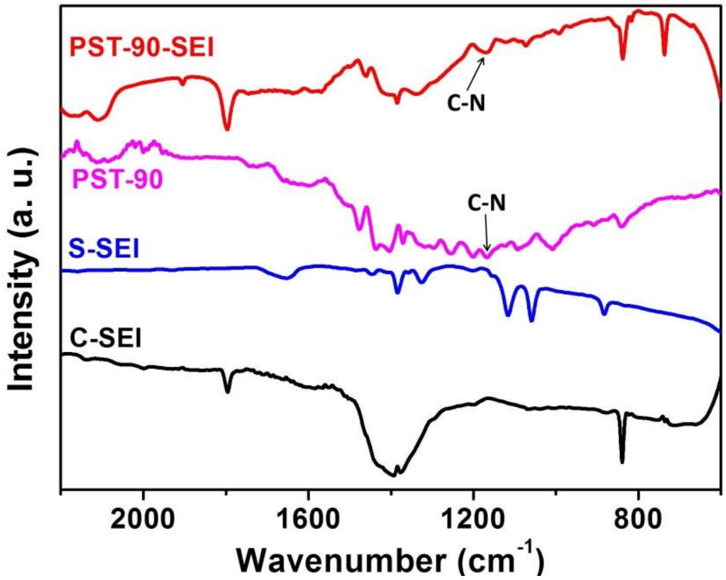

Supplementary Figure 4. FT-IR of SEI layers obtained from the control electrolyte (C-SEI), the S-Electrolyte (S-SEI) and the PST-90-Electrolyte (PST-90-SEI). The data indicates the existence of organosulfide/organopolysulfide originating from the PST-90 in the SEI layer.

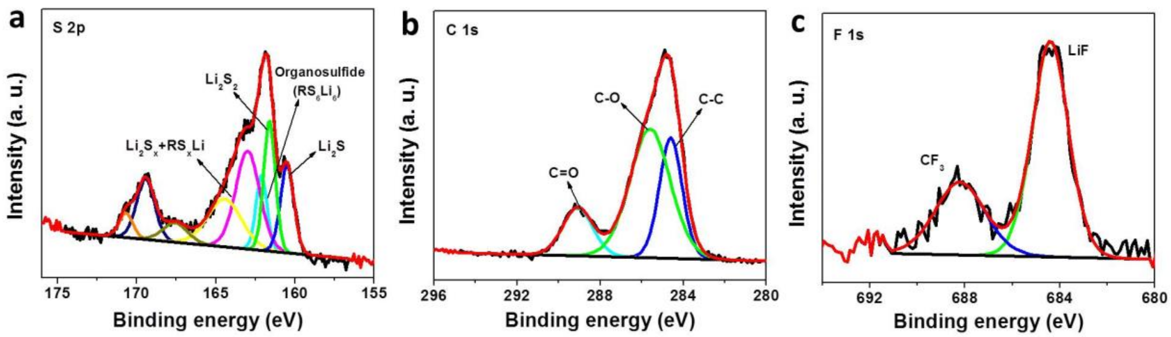

Supplementary Figure 5. S 2*p* XPS spectra (a), C 1*s* XPS spectra (b), and F 1*s* XPS spectra (c) of SEI layers obtained at fully Li plating state. These results are similar to those of SEI layers obtained at fully Li stripping state, which indicate as-formed hybrid SEI layer is stable at both Li plating and stripping states.

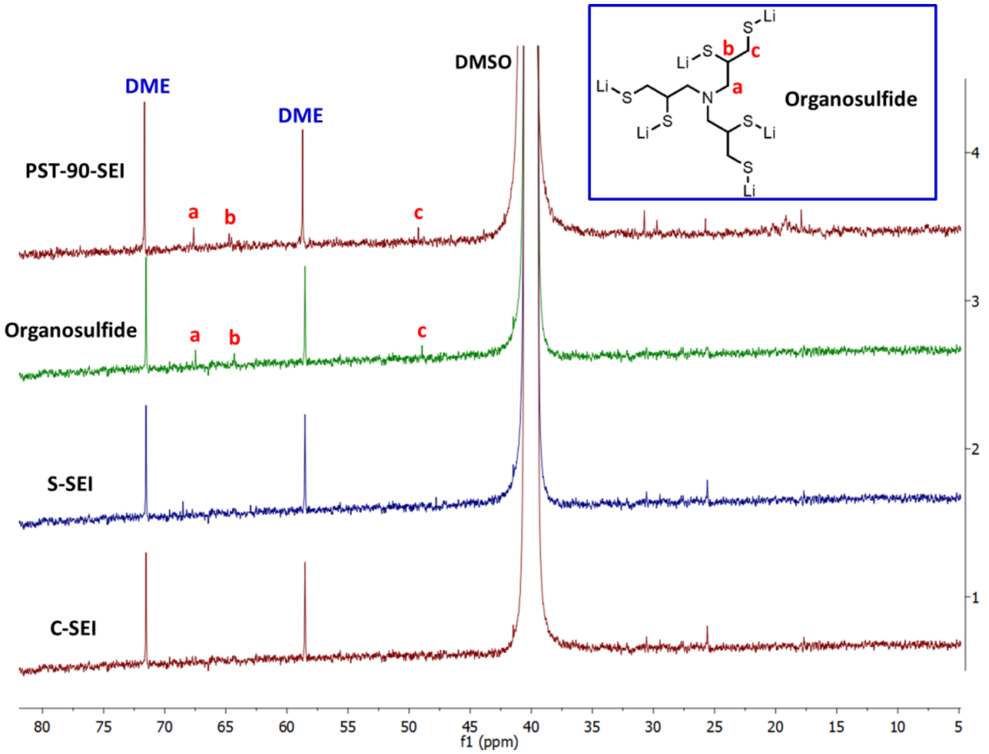

Supplementary Figure 6. ^{13}C NMR spectra of C-SEI, S-SEI and PST-90-SEI. The same amount of SEI was dispersed in DMSO-d6 (~10 mg SEI in 0.5 ml DMSO-d6) for NMR characterization. The organic units in SEI layers dissolved in the DMSO-d6 can be detected; the insoluble components are ^{13}C NMR silent. Organosulfide (RS_6Li_6) was synthesized from the reaction between PST-90 and Li_2S at a specific ratio in DME (Li polysulfides were also formed during this reaction, because Li polysulfides are ^{13}C NMR silent, only constant peaks from organosulfide can be observed). All the SEI layers were obtained after 100 cycles of Li plating/stripping, and Li was stripped completely. All the SEI layers were washed with DME before the characterization.

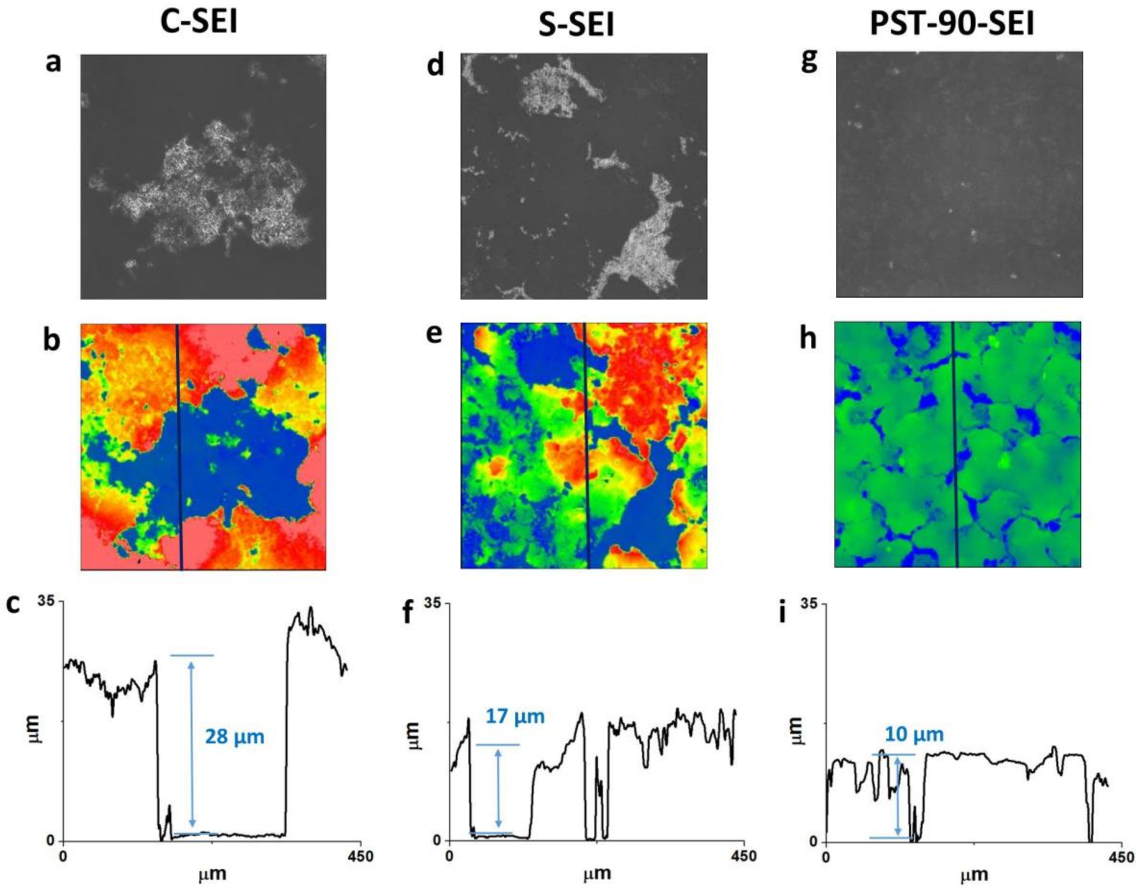

Supplementary Figure 7. Thickness and coverage degree of SEI layers formed from the different electrolytes. (a, d, g) Optical microscope images of C-SEI, S-SEI and PST-90-SEI layer. (b, e, h) Optical profilometry images of C-SEI, S-SEI and PST-90-SEI layer. (c, f, i) Cross-section line profile of C-SEI, S-SEI and PST-90-SEI layer. The plot in cross-section line profile corresponds to the black line in optical profilometry image. The height from valley to high plateau (marked in (c, f, i)) represents the average film thickness of the SEI layers. The results show the thicknesses of C-SEI, S-SEI and PST-90-SEI layer are 28, 17 and 10 μm, respectively. The coverage degrees of C-SEI, S-SEI and PST-90-SEI layer are 50, 70 and 95%, respectively. All the SEI layers were obtained after 100 cycles of Li plating/stripping, and Li was stripped completely before the characterization. The scan size was 450 μm×450 μm.

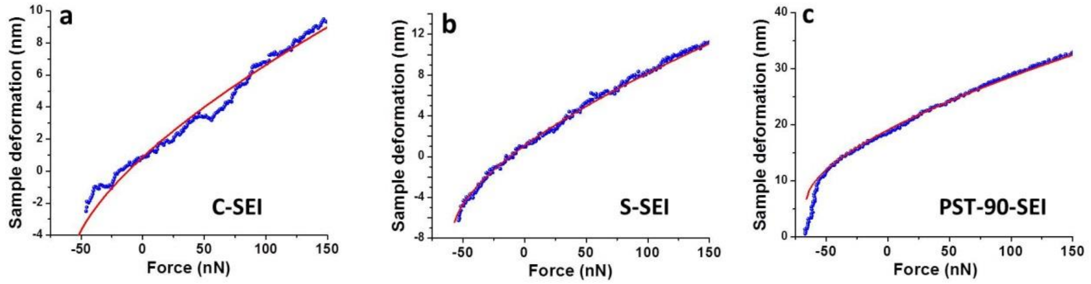

Supplementary Figure 8. JKR model fit of the unloading curves of SEI layers formed from the control electrolytes (C-SEI) (a), the S-Electrolyte (S-SEI) (b), and the PST-90-Electrolyte (PST-90-SEI) (c).

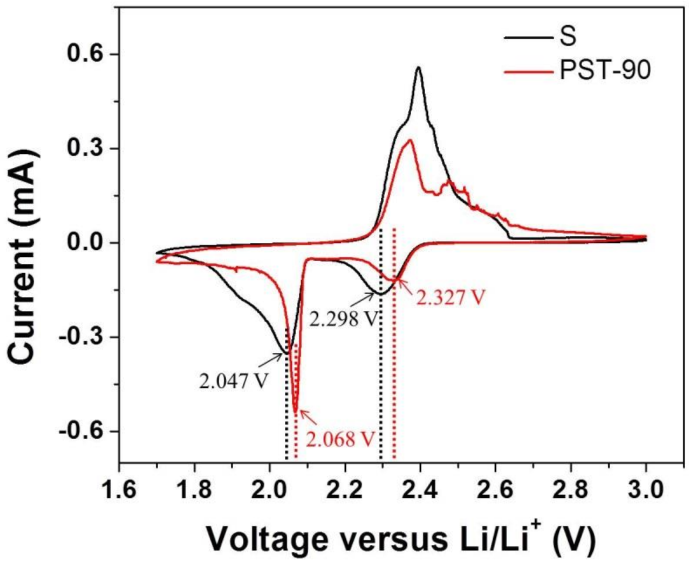

Supplementary Figure 9. Cyclic voltammetry (CV) curves of sulfur and PST-90. The CV curves show PST-90 has higher reduction potential than that of sulfur, which indicates PST-90 reacts with Li metal much easier and faster than sulfur. CV curves were collected at a scan rate of 0.02 mV s^{-1} from 3.0 to 1.7 V.

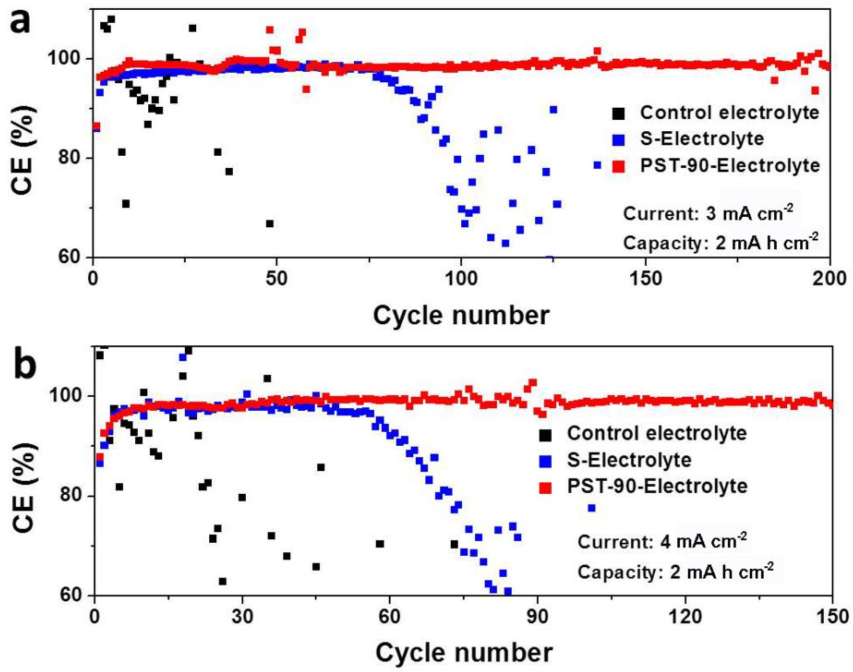

Supplementary Figure 10. Cycling performance of cells using electrolyte with different additives at a deposition capacity of 2 mA h cm^{-2} and current densities of 3 mA cm^{-2} (a) and 4 mA cm^{-2} (b).

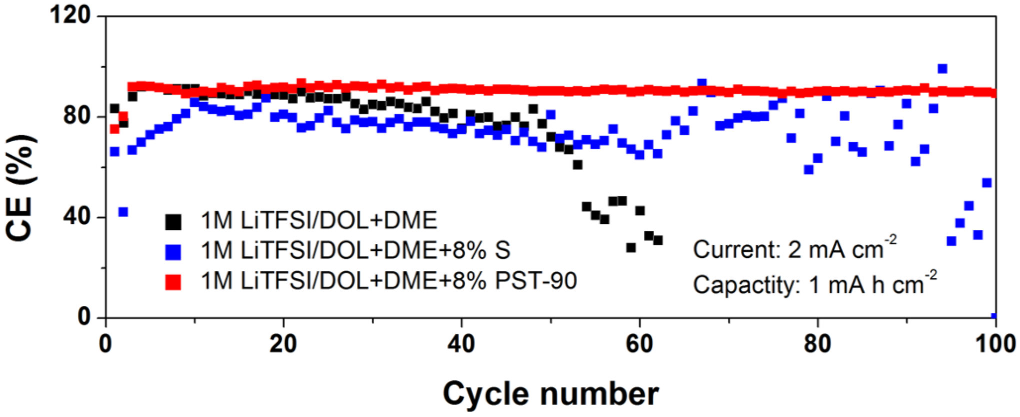

Supplementary Figure 11. Cycling performance of cells with electrolytes only using sulfur-containing compounds at a current density of 2 mA cm^{-2} and a deposition capacity of 1 mA h cm^{-2}.

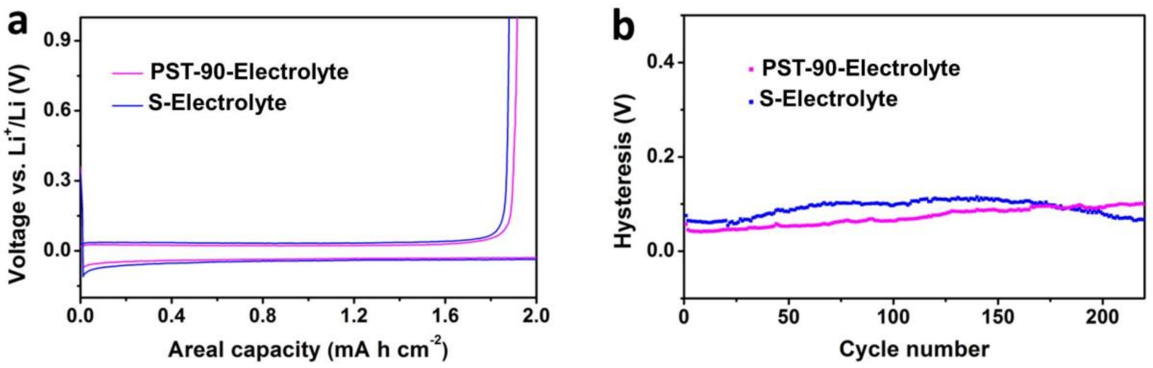

Supplementary Figure 12. Voltage profiles and average voltage hysteresis of the cells using electrolyte containing different additives. (**a**) Voltage profiles and (**b**) average voltage hysteresis of the Li plating/stripping process with Li metal as the reference/counter electrode at a current density of 2 mA cm^{-2} with a deposition capacity of 2 mA h cm^{-2} using S-Electrolyte (blue symbols) and PST-90-Electrolyte (magenta symbols). The voltage profiles are obtained at the first cycle of Li plating/striping.

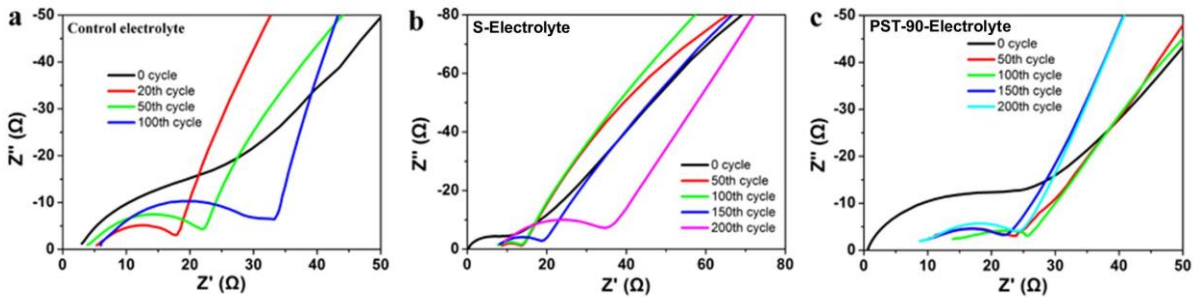

Supplementary Figure 13. The EIS curves of the cells using different electrolytes after different cycles of Li plating/stripping. (**a**) The cell using the control electrolyte. (**b**) The cell using the S-Electrolyte. (**c**) The cell using the PST-90-Electrolyte. The Li was completely stripped before the EIS characterization.

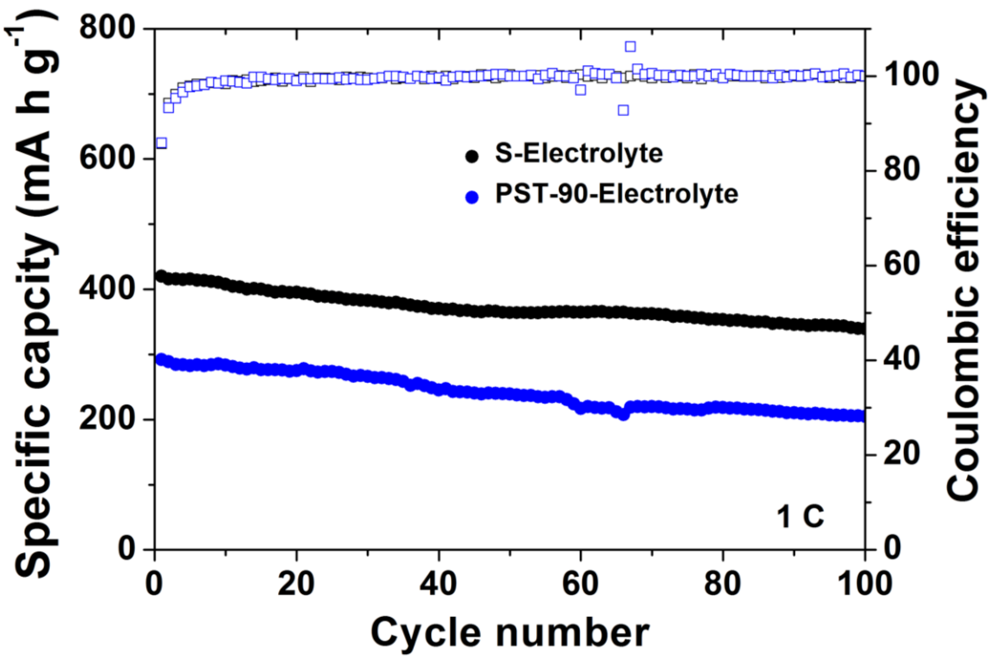

Supplementary Figure 14. Cycling performance of a sulfur-free carbon cathode using the S-Electrolyte or PST-90-Electrolyte at a current density of 1 C. The initial capacities are 420 and 292 mA h/g for the S-Electrolyte and PST-90-Electrolyte, respectively. The capacity was calculated based on the mass of sulfur.

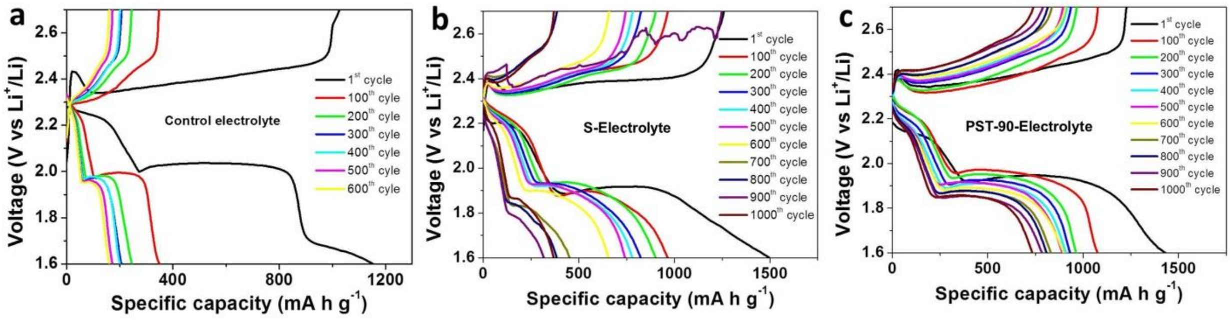

Supplementary Figure 15. Discharge-charge profiles of Li-S batteries using electrolytes containing different additives at a rate of 1 C. (**a**) The control electrolyte. (**b**) The S-Electrolyte. (**c**) The PST-90-Electrolyte.

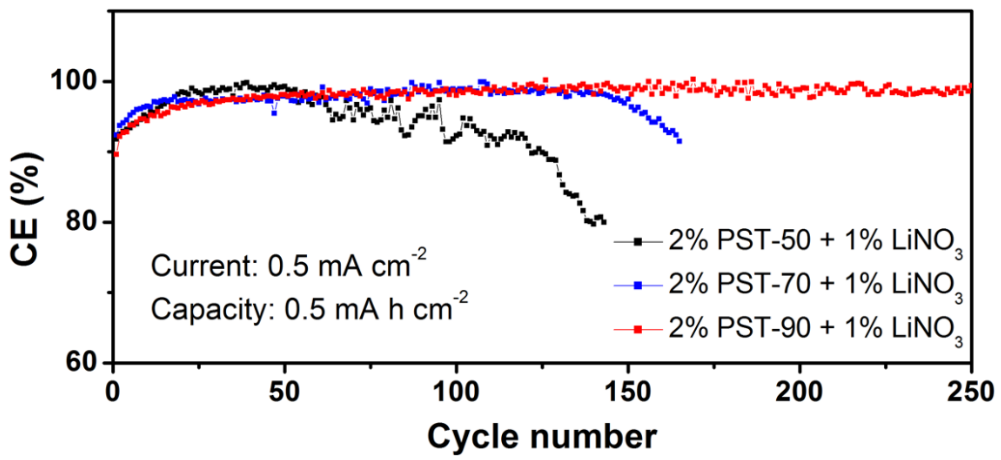

Supplementary Figure 16. Cycling performance of 2 wt% PST containing different sulfur contents as additives. The cycling performance shows that PST with 90 wt% sulfur (PST-90) exhibits the best cycling performance, and the cycling stability drops with the decrease of sulfur content in the PST.

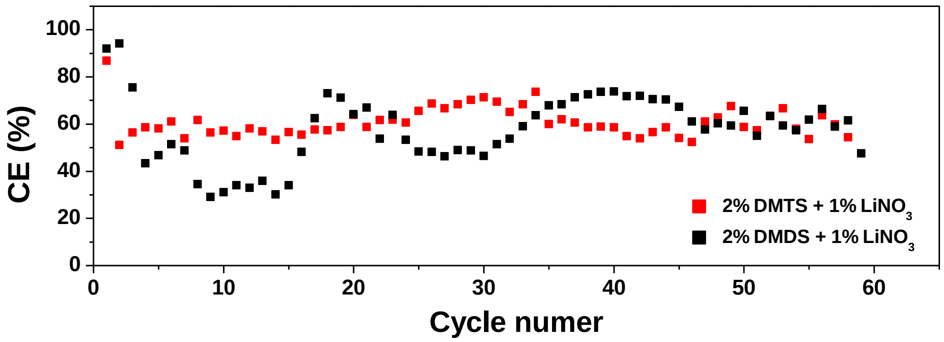

Supplementary Figure 17. The CE versus cycle number of cells containing electrolytes with 2 wt% DMDS or DMTS as additive at the current density of 0.5 mA cm^{-2} and deposition capacity of 0.5 mA h cm^{-2}. The data shows that the cells with electrolytes containing DMDS or DMTS as additive show very poor cycling stability and low CE, which indicates DMDS and DMTS could not enable the formation of stable SEI layer.

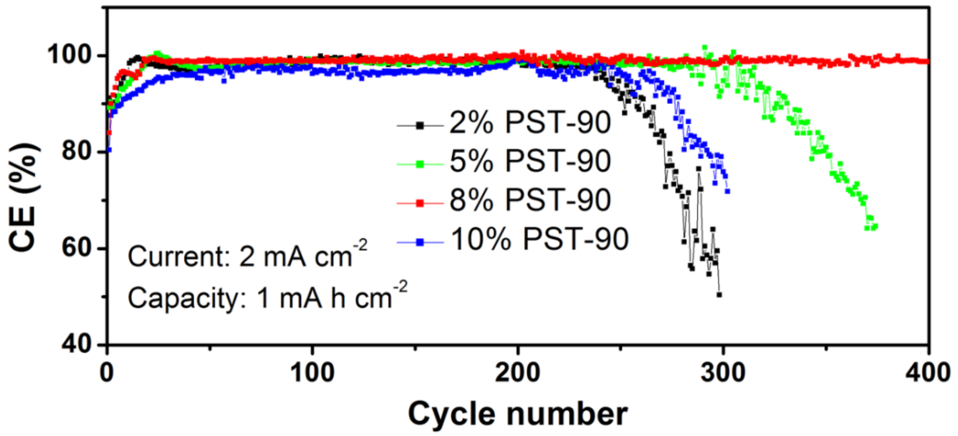

Supplementary Figure 18. The CE versus cycle number of cells containing electrolyte with different contents of PST-90. The data shows that the electrolytes with 8 wt% PST-90 show the best cycling life.

Supplementary Table 1. The reduced modulus of SEI layers formed from the control electrolyte, the S-Electrolytes and the PST-90-Electrolyte.

**Electrolyte**	**SEI layer**	**Reduce modulus (JKR model)/MPa**
Control electrolyte	Inorganic layer (C-SEI)	903
S-Electrolyte	Inorganic layer (S-SEI)	740
PST-90-Electrolyte	Inorganic/organic layer (PST-90-SEI)	367

Supplementary Table 2. Prepared SCPs with different contents of sulfur.

Samples	Content (wt%)	
	Sulfur	TAA
PST-50	50	50
PST-70	70	30
PST-90	90	10

Supplementary Table 3. Summary of Li plating/stripping CE of cells using ether-based electrolytes.

Strategy	Electrolyte	CE at different deposition capacities and current densities			Reference
		1 mA h cm^{-2}	2 mA h cm^{-2}	3 mA h cm^{-2}	
Interfacial layer (Interconnected hollow carbon nanospheres)	1 M LiTFSI/DOL + DME + 2% $LiNO_3$ + 0.1 M Li_2S_8	~99% (1 mA cm^{-2}, 150 cycles)	N/A	N/A	1
3D current collector	1 M LiTFSI/DOL + DME	~97% (0.5 mA cm^{-2}, 50 cycles)	N/A	N/A	2
Additive	1 M LiTFSI/DOL + DME + 5% $LiNO_3$ + 0.18 M Li_2S_8	~99% (2 mA cm^{-2}, 400 cycles)	~98.5% (2 mA cm^{-2}, 200 cycles), N/A at both 3 mA cm^{-2} and 4 mA cm^{-2})	~98.5% (2 mA cm^{-2}, 200 cycles)	3
Highly concentrated electrolyte	4 M LiFSI/DME	98.4% (0.5 mA h cm^{-2}, 4 mA cm^{-2}, 1000 cycles)	N/A	N/A	4
3D oxidized polyacrylonitrile nanofiber layer	1 M LiTFSI/DOL + DME + 2% $LiNO_3$	97.9% (1 mA cm^{-2}, 120 cycles); 97.4% (3 mA cm^{-2}, 120 cycles)	N/A	N/A	5
Glass fiber	1 M LiTFSI/DOL + DME + 2% $LiNO_3$	97% (0.5 mA h cm^{-2}, 1 mA cm^{-2}, 65 cycles)	N/A	N/A	6
3D current collector	1 M LiTFSI/DOL + DME + 1% $LiNO_3$	97% (1 mA cm^{-2}, 140 cycles)	N/A	N/A	7
Soft polymer coating	1 M LiTFSI/DOL + DME + 1% $LiNO_3$	97% (1 mA cm^{-2}, 180 cycles)	N/A	97.7% (1 mA cm^{-2}, 80 cycles)	8
Interfacial layer (PDMS film)	1 M LiTFSI/DOL + DME + 1% $LiNO_3$	98.2% (1 mA cm^{-2}, 100 cycles)	N/A	N/A	9

**Additive (Our work)**	**PST-90-Electrolyte**	**99% (2 mA cm^{-2}, 400 cycles)**	**98.9% (2 mA cm^{-2}, 220 cycles) 98.7% (3 mA cm^{-2}, 200 cycles) 98.6% (4 mA cm^{-2}, 150 cycles)**	**98.6% (2 mA cm^{-2}, 220 cycles)**	**Our work**

Supplementary Note 1.

The reduced modulus of the SEI layers was estimated from nanoindentation tests.[10-14] Because of the strong adhesion behavior for the sample we investigated, the Johnson-Kendall-Roberts (JKR) fitting equation has been previously derived and applied to analyze the soft materials.[12, 15] D.M. Ebenstein et al. already provided the simplified derivation of the fitting equation, which can be applied to our experiments.[15] Supplementary Fig. 3 displays the JKR fitting results of the indentation tests. The Poisson ratio of SEI film still remained unknown, and only a rough range was given (0.27~0.5) in some studies.[16, 17] As alternative, the reduced elastic modulus was able to be calculated directly from JKR fitting equation and was shown in Supplementary Table 1.

Supplementary Methods

General procedure for the preparation of PST. To a 24 ml glass vial equipped with a magnetic stir bar was added sulfur and the vial was then heated to 145 ^{o}C in a thermostatted oil bath until a clear orange-colored molten phase was formed. TAA was then added directly to the molten sulfur medium via a syringe. The resulting mixture was stirred at 145 ^{o}C for 8–10 minutes, which resulted in vitrification of the reaction medium. After allowing the reaction mixture to cool to room temperature, a brown solid (PST) was formed.

Preparation of PST-90 (90 wt% S). The copolymerization was carried out by following the general procedure written above with S_8 (4.50 g, 17.6 mmol) and TAA (0.50 g, 3.64 mmol) to afford a brown solid (yield: 4.90 g).

Preparation of PST-70 (70 wt% S). The copolymerization was carried out by following the general procedure written above with S_8 (3.50 g, 13.7 mmol) and TAA (1.50 g, 10.9 mmol) to afford a brown solid (yield: 4.87 g).

Preparation of PST-50 (50 wt% S). The copolymerization was carried out by following the general procedure written above with S_8 (2.50 g, 9.77 mmol) and TAA (2.50 g, 18.2 mmol) to afford a brown solid (yield: 4.62 g).

Supplementary References.

1. Zheng, G., *et al.* Interconnected hollow carbon nanospheres for stable lithium metal anodes. *Nat. Nanotechnol.* **9**, 618-623 (2014).
2. Yang, C. P., *et al.* Accommodating lithium into 3D current collectors with a submicron skeleton towards long-life lithium metal anodes. *Nat. Commun.* **6**, 8058 (2015).
3. Li, W., *et al.* The synergetic effect of lithium polysulfide and lithium nitrate to prevent lithium dendrite growth. *Nat. Commun.* **6**, 7436 (2015).
4. Qian, J., *et al.* High rate and stable cycling of lithium metal anode. *Nat. Commun.* **6**, 6362 (2015).
5. Liang, Z., *et al.* Polymer nanofiber-guided uniform lithium deposition for battery electrodes. *Nano Lett.* **15**, 2910-2916 (2015).
6. Cheng, X.-B., *et al.* Dendrite-free lithium deposition induced by uniformly distributed lithium ions for efficient lithium metal batteries. *Adv. Mater.* **28**, 2888-2895 (2016).
7. Yun, Q. B., *et al.* Chemical dealloying derived 3D porous current collector for Li metal anodes. *Adv. Mater.* **28**, 6932-6939 (2016).
8. Zheng, G. Y., *et al.* High-performance lithium metal negative electrode with a soft and flowable polymer coating. *ACS Energy Lett.* **1**, 1247-1255 (2016).
9. Zhu, B., *et al.* Poly(dimethylsiloxane) thin film as a stable interfacial layer for high-performance lithium-metal battery anodes. *Adv. Mater.* **29**, 1603755-1603761 (2017).
10. Opdahl, A., *et al.* Surface mechanical properties of pHEMA contact lenses: Viscoelastic and adhesive property changes on exposure to controlled humidity. *J. Biomed. Mater. Res., Part A* **67A**, 350-356 (2003).

11. Cappella, B., Kaliappan, S. K., Sturm, H. Using AFM force-distance curves to study the glass-to-rubber transition of amorphous polymers and their elastic-plastic properties as a function of temperature. *Macromolecules* **38**, 1874-1881 (2005).

12. He, X., Barthel, A. J., Kim, S. H. Tribochemical synthesis of nano-lubricant films from adsorbed molecules at sliding solid interface: Tribo-polymers from alpha-pinene, pinane, and n-decane. *Surf. Sci.* **648**, 352-359 (2016).

13. Cappella, B., Dietler, G. Force-distance curves by atomic force microscopy. *Surf. Sci. Rep.* **34**, 1-104 (1999).

14. Cappella, B., Silbernagl, D. Nanomechanical properties of polymer thin films measured by force-distance curves. *Thin Solid Films* **516**, 1952-1960 (2008).

15. Ebenstein, D. M., Wahl, K. J. A comparison of JKR-based methods to analyze quasi-static and dynamic indentation force curves. *J. Colloid Interface Sci.* **298**, 652-662 (2006).

16. Kuznetsov, V., *et al.* Wet nanoindentation of the solid electrolyte interphase on thin film Si electrodes. *ACS Appl. Mater. Interfaces* **7**, 23554-23563 (2015).

17. Greaves, G. N., Greer, A. L., Lakes, R. S., Rouxel, T. Poisson's ratio and modern materials. *Nat. Mater.* **10**, 823-837 (2011).
